# Genetic array data from the Northern Ireland COhort for the Longitudinal study of Ageing (NICOLA)

**DOI:** 10.21203/rs.3.rs-9075179/v1

**Published:** 2026-05-06

**Authors:** Laura Smyth, Marisa Canadas Garre, Claire Hill, Claire Potter, Ian Young, Bernadette McGuinness, Frank Kee, Amy Jayne McKnight

**Affiliations:** Queen's University Belfast; Queen's University Belfast; Queen's University Belfast; Queen's University Belfast; Queen's University Belfast; Queen's University Belfast; Queen's University Belfast; Queen's University Belfast

**Keywords:** Ageing, Association, Genetic, Longitudinal, Molecular, NICOLA, Northern Ireland, Population, SNP

## Abstract

Northern Ireland’s largest national population cohort over 50 years of age, with a focus on the collection of longitudinal data including computer-assisted personal interviews, self-completed questionnaires and in-person health assessments takes form as the Northern Ireland COhort for the Longitudinal study of Ageing (NICOLA). Data from 8,283 individuals over 50 years of age was collected from individuals within NICOLA’s first wave of data collection for this cohort. Together, the data collected aims to help researchers explore how social, economic, health, lifestyle, and biological factors influence healthy ageing. NICOLA places a strong emphasis on molecular biomarkers, with a subset of 3,471 participants (44%) undergoing health assessments at wave 1, including the collection of blood samples specifically for this analysis.

**Objectives:**

Using blood-derived DNA collected from individuals within NICOLA, genetic data was generated to enable research projects associated with rich cross-sectional and longitudinal data within NICOLA to explore genetic connections to the health, lifestyle, environment and financial situations of people as they grow older.

**Data description:**

Genetic data was generated using the Illumina Infinium CoreExome-24 array (Illumina, USA) and is available for 2,978 NICOLA participants with 551,839 directly genotyped and 18,148,478 imputed single nucleotide polymorphisms following initial quality control.

## Objective

Northern Ireland’s largest population cohort collecting longitudinal data from a sample of the over-50s population (n=8,283) was launched in 2012 as the Northern Ireland Cohort for the Longitudinal Study of Ageing (NICOLA) [1], [2]. Throughout the recruitment, spouses or partners of invited individuals who resided in the same home at time of interview, who were under the age of 50 (n=195, n=8,478 total population) were also invited to take part. A strong focus was placed on the generation of molecular biomarkers as part of the Wave 1 data collection and analysis. The genetic data generated forms a key part of this and is described in this data note.

Genetic data was generated using the Illumina Infinium CoreExome-24 array (Illumina, USA) for 3,266 individuals and is available for 2,978 NICOLA participants following initial quality control for 551,839 directly genotyped and 18,148,478 imputed single nucleotide polymorphisms (SNPs). Summary statistics for the association of these gene polymorphisms with ~30 phenotypes have been generated to date and are available as derived variables from the NICOLA Data Access Committee[3].

The genetic .bam, .bim and .fam data files are available through data access requests made to the European Genome-Phenome Archive using study ID EGAS00001007915, dataset ID EGAD00010002762.

## Data description

### NICOLA Sample Collection and Preparation

NICOLA participants who consented to the in-person health assessment element of data collection provided blood samples which were collected in EDTA tubes at the clinical research facility of Queen’s University Belfast. Sample processing using aseptic technique was conducted in laboratories on the same floor of the facility; blood tubes were centrifuged at 3000 rpm for 10 minutes at 4°C before a 1 mL pipette was used to carefully remove each buffy coat before being frozen at −80°C. DNA was extracted, quantified using PicoGreen, normalised to 200 ng/μL using 0.1 TE and stored in aliquots at −80°C to minimise freeze/thaw cycles. Critically important for this data, all samples were processed in a single recruitment laboratory with genetic data generated in a single laboratory. All wet-lab typing included standard experimental controls including replicate samples (n=12), control samples of known genotype blinded to the genotyping laboratory (n=7) and no template controls (n=4).

### Measurement of Genetic Biomarkers

Samples were genotyped by Eurofins Scientific (https://www.eurofinsgenomics.eu). Genotype data (n=551,839 markers directly typed) was generated using the Illumina Infinium CoreExome-24 on an iScan for two batches composed of 2,799 and 467 participants respectively (n=3,266). For the purposes of analysis, each batch was processed separately as they were genotyped at different timepoints.

GenomeStudio^®^ Software’s Genotyping Module was used alongside the Genome Reference Consortium Human Build 37 (GRCh37) for analysis. Each SNP was called using clustering methods with an independent visual review of any outliers to more accurately call genotypes. Samples were excluded if they had insufficient DNA quality, quantity, or poor genotype concordance with previous genotypes during careful evaluation.

### Quality Control and Imputation of Genetic Data

The QC of genotyped data was performed in PLINK 1.90 beta[4]. QC included removal of samples with a call rate <95%, heterozygosity, principal component analysis outliers, sex mismatches and duplicates. Up to second-degree related individuals were also removed by eliminating one individual from each pair with an identity by descent value >0.1875[5], [6]. Variants with a call rate ≥98%, Hardy-Weinberg equilibrium p>10^−6^ and minor allele frequency <0.0001 were removed.

Files were prepared for imputation using the Haplotype Reference Consortium (HRC)*/* 1000 Genomes *Imputation Preparation and Checking Tool*, developed by Will Rayner[7], before imputation to the 1000 Genomes Phase3 v5 (1KGP3) and HRC r1.1 2016 reference panels using the Michigan Imputation Server[8]. A minor allele cut-off of 5 and imputation quality of 0.3 was applied to imputed files; monomorphic markers were removed.

Upon completion of initial QC, 2,526 samples for batch 1 and 452 samples for batch 2 remained in the cohort (n=2,978 total). An overview of the datafiles is included in Table 1.

### Exemplar Genetic Data

GWAS analysis of the NICOLA dataset has contributed to an international GWAS examining human height[9], an international multi-ancestry, genome-wide genetic discovery meta-analysis of lipid levels[10], [11], [12] and two GWAS examining age-related macular degeneration[13], [14]. The associated summary statistics have been returned to the NICOLA bioresource and are available for researchers within our data access agreements.

## Limitations

- Genetic data is not available for all individuals who participated in NICOLA, rather is available for a sub-set of the larger cohort from Wave 1. All individuals who attended the health assessment and provided a venous blood sample with permission for genetic analysis were submitted for genotyping.
- Despite being a nationally representative cohort, there is a limitation in terms of the response bias which has an over-representation of individuals with higher education levels.
- Despite the study being designed to be representative of the over 50s population in NI using a random selector for participants based on residential address, there is potential for selection bias to have attracted more healthy individuals.
- Due to the small number of ethnic minorities included within NICOLA, no statistically robust estimates could be generated for this population subset.

## Figures and Tables

**Table 1:** Overview of data files/data sets.

Label	Name of data file/data set	File types (file extension)	Data repository and identifier (DOI or accession number)
Data file 1	Genetic data .bed file for batch 1 of the Northern Ireland Cohort for the Longitudinal Study of Ageing (NICOLA)	.bed	EGA ID: EGAD00010002762 DAC ID: EGAC00001003504
Data file 2	Genetic data .bim file for batch 1 of NICOLA	.bim	EGA ID: EGAD00010002762 DAC ID: EGAC00001003504
Data file 3	Genetic data .fam file for batch 1 of NICOLA	.fam	EGA ID: EGAD00010002762 DAC ID: EGAC00001003504
Data file 4	Genetic data .bed file for batch 2 of NICOLA	.bed	EGA ID: EGAD00010002762 DAC ID: EGAC00001003504
Data file 5	Genetic data .bim file for batch 2 of NICOLA	.bim	EGA ID: EGAD00010002762 DAC ID: EGAC00001003504
Data file 6	Genetic data .fam file for batch 2 of NICOLA	.fam	EGA ID: EGAD00010002762 DAC ID: EGAC00001003504

## Data Availability

The data described in this Data note can be freely and openly accessed through the European Genome-Phenome Archive under the study accession number EGAS00001007915.

## Abbreviations

1KGP3: 1000 Genomes Phase3 v5
DNA: deoxyribose nucleic acid
EDTA: ethylenediaminetetraacetic acid
GRCh37: Genome Reference Consortium Human Build 37
HRC: Haplotype Reference Consortium
NICOLA: Northern Ireland COhort for the Longitudinal study of Ageing
SNP: single nucleotide polymorphism
QC: quality control
